# Integration of Shear-Wave Elastography and Inertial Motion Sensing for Quantitative Monitoring of Tendon Remodeling After Shockwave Therapy in Greater Trochanteric Pain Syndrome

**DOI:** 10.3390/bioengineering13010083

**Published:** 2026-01-12

**Authors:** Gabriele Santilli, Antonello Ciccarelli, Francesco Agostini, Andrea Bernetti, Mario Vetrano, Sveva Maria Nusca, Eleonora Latini, Massimiliano Mangone, Samanta Taurone, Daniele Coraci, Giorgio Felzani, Marco Paoloni, Valter Santilli

**Affiliations:** 1 Department of Movement, Human and Health Sciences, Division of Health Sciences, University of Rome “Foro Italico”, 00135 Rome, Italy; 2 Department of Anatomical and Histological Sciences, Legal Medicine and Orthopedics, Sapienza University, 00185 Rome, Italy; 3 IRCCS San Raffaele Roma, Via della Pisana 235, 00163 Rome, Italy; 4 Department of Biological and Environmental Science and Technologies, University of Salento, 73100 Lecce, Italy; 5 Physical Medicine and Rehabilitation Unit, Sant’Andrea Hospital, Sapienza University of Rome, 00189 Rome, Italy; 6 Rehabilitation Unit, Department of Neuroscience, University of Padova, 35122 Padova, Italy; 7 San Raffaele Sulmona, Viale dell’Agricoltura, 67039 Sulmona, Italyvalter.santilli@sanraffaele.it (V.S.)

**Keywords:** gluteus medius tendinopathy, greater trochanteric pain syndrome, extracorporeal shockwave therapy, biomarkers, biosensors, shear wave elastography, tendon thickness, SWE velocity, pain assessment, functional outcome, chronic tendinopathy, hip abduction

## Abstract

Background: Greater trochanteric pain syndrome (GTPS) is associated with structural tendon alterations and functional impairment. Extracorporeal shockwave therapy (ESWT) is a common treatment, but objective monitoring of tendon remodeling and motor recovery remains limited. Objective: This study aimed to integrate shear-wave elastography (SWE) expressed in m/s and wearable inertial measurement unit (IMU) as biosensing tools for the quantitative assessment of tendon elasticity, morphology, and hip motion after ESWT in GTPS. Methods: In a prospective cohort of adults with chronic GTPS, shear wave elastography (SWE) quantified gluteus medius tendon (GMT) elasticity and thickness, while hip abduction range of motion (ROM) was measured using a triaxial inertial measurement unit. Clinical scores on the Visual Analogue Scale (VAS), Harris Hip Score (HHS), Low Extremity Functional Scale (LEFS), and Roles and Maudsley score (RM) were collected at baseline (T0) and at 6 months (T1). Results: Thirty-five patients completed follow-up. Pain and function improved significantly (VAS, HHS, LEFS, RM; all *p* < 0.05). SWE values of the affected GMT increased, while tendon thickness decreased yet remained greater than on the contralateral side. Hip abduction ROM increased significantly from T0 to T1 (*p* < 0.05). Correlation analysis showed a negative association between abduction and pain at T1 (r = −0.424; *p* = 0.011) and, at baseline, between abduction and VAS (r = −0.428; *p* = 0.010) and RM (r = −0.346; *p* = 0.042), and a positive association with LEFS (r = 0.366; *p* = 0.031). SWE correlated negatively with VAS at T1 (r = −0.600; *p* < 0.05) and positively with HHS at T1 (r = 0.400; *p* < 0.05). Conclusions: Integrating elastography with inertial sensor-based motion analysis provides complementary, quantitative insights into tendon remodeling and functional recovery after ESWT in GTPS. These findings support combined imaging and wearable motion measures to monitor treatment response over time.

## 1. Introduction

Gluteal tendinopathy (GT) is a leading cause of lateral hip pain frequently encountered in primary care settings [1]. Patients with severe GT often experience a significantly reduced quality of life, comparable to those with end-stage hip osteoarthritis, along with similarly high levels of pain and functional impairment [2]. This condition can severely hinder physical function, leading to a lower likelihood of full-time employment compared to both asymptomatic individuals and those diagnosed with hip osteoarthritis [3]. Patients with GT tend to exhibit a higher body mass index (BMI), decreased pain thresholds, weakened hip abduction strength, and elevated levels of anxiety and depression relative to healthy controls [4,5,6]. The profound impact of GT on overall quality of life has been underscored by studies examining a broad range of biopsychosocial factors [6].

The primary anatomical sources of GT is the tendinopathy of the gluteus medius (GMTp) [7,8,9,10]. The term “greater trochanteric pain syndrome” (GTPS) is often used interchangeably with GMTp [11]. GTPS most commonly affects individuals in their 40s to 60s and shows a marked gender bias, with women comprising approximately 73% of cases [2,12]. Common clinical features include difficulty sleeping on the affected side, symptom aggravation with stair climbing, and inability to engage in sports or recreational activities [13,14]. Tendinopathies can lead to a reduction in joint range of motion (ROM), particularly in movements involving the muscle affected by the tendinopathy [15,16]. In particular, individuals with GTPS have been shown to exhibit weaker hip abduction compared to age-matched healthy adults [4,5,17].

Ultrasound (US) is a first-choice imaging modality for diagnosing GTPS [18]. Recent research has explored various treatment approaches, including corticosteroid and platelet-rich plasma injections [19,20], education programs [14] as well as extracorporeal shockwave therapy (ESWT) [21] with promising outcomes reported. In recent years, ESWT has gained recognition as an effective and safe approach to managing tendinopathy [22,23,24,25]. The increasing adoption of ESWT is attributed to multiple advantages: high clinical success rate [26], the need for only a few treatment sessions, short therapy duration, cost-effectiveness, greater accessibility, and minimal restrictions to daily activities. However, despite treatment, some patients may continue to experience persistent symptoms or recurrence of tendinopathy, which can significantly diminish their quality of life [27].

Conducting a more in-depth evaluation based on objective diagnostic findings, rather than solely on subjective assessment, may be crucial for informing clinical decision-making. From this standpoint, our study evaluated the gluteus medius tendon stiffness through elastosonography, in combination with tendon thickness measurements, to offer a more comprehensive assessment of these patients with GTPS. Despite the growing interest in imaging biomarkers, no previous study has simultaneously and quantitatively evaluated shear wave elastography (SWE) (m/s) and tendon thickness as structural outcome measures after ESWT.

This prospective cohort study was conducted to evaluate the clinical, ultrasonographic, and functional effects of ESWT in patients with GTPS. SWE expressed in (m/s) was used to quantitatively assess tendon elasticity and thickness, alongside validated clinical outcome measures including the Harris Hip Score (HHS), Lower Extremity Functional Scale (LEFS), Roles and Maudsley scale (RM), and Visual Analogue Scale (VAS). In addition, active hip abduction ROM was measured using an inertial measurement unit (IMU) to capture potential changes in mobility. By integrating structural imaging, quantitative motion analysis, and clinical assessment, this study aimed to investigate the correlation between morphological tendon changes and functional recovery following ESWT. Patients were prospectively enrolled prior to treatment and followed for 6 months, with SWE (m/s), ROM measurement, and clinical outcome scores collected at baseline and at the end of follow-up.

## 2. Materials and Methods

The study protocol received approval from the Institutional Review Board of La Sapienza University (Protocol No. 0000/2024, approved on 14 February 2024) before the initiation of the research, as it involved human participants. Patients did not participate in defining the study design. All participants provided written informed consent prior to enrollment. The study has been conducted and reported in accordance with the STROBE guidelines.

### 2.1. Study Design and Population

This investigation was designed as a prospective, single-center cohort study carried out at our tertiary academic medical facility in Rome, Italy, between April 2024 and May 2025. Prior to enrollment, all participants provided written informed consent, authorizing the use of their anonymized data for research purposes in compliance with privacy regulations. Consecutive patients diagnosed with GTPS who satisfied the inclusion criteria and agreed to participate were recruited until the required sample size was achieved. Each participant was prospectively monitored for six months following completion of ESWT. The main outcome measure was clinical improvement, evaluated through the HHS and ultrasonographic assessment of the gluteus medius tendon (GMT). Eligibility criteria included (1) age ≥ 18 years, (2) presence of unilateral lateral hip pain lasting at least three months, (3) discomfort when lying on the symptomatic side, (4) tenderness upon palpation of the greater trochanter, and (5) signed informed consent. Exclusion criteria were (1) tendon rupture, (2) prior hip surgery, (3) radiographic evidence of advanced hip osteoarthritis (Kellgren–Lawrence grade ≥ 3), (4) chronic lumbar pain, (5) systemic vascular, neurological, or rheumatologic disease, (6) malignancy or local infection, (7) pregnancy, (8) coagulopathy or ongoing anticoagulant therapy, and (9) previous conservative interventions for GTPS within the last three months. At baseline, demographic and clinical data (age, sex, body mass index) were recorded. Functional and pain assessments (HHS, VAS, LEFS, RM), hip abduction range of motion (ROM), and sonographic parameters (GMT thickness and shear-wave elastography velocity) were collected both before treatment (T0) and six months after the final ESWT session (T1).

### 2.2. Intervention

The treatment protocol adhered to current best practices for managing GTPS with ESWT. Procedures were carried out by the principal investigators using the Modulith SLK system (Storz Medical, Tägerwilen, Switzerland), an electromagnetic shockwave device equipped with an integrated ultrasound targeting system for precise localization of the treatment area. Patients were positioned in the lateral decubitus posture, and a coupling medium consisting of ultrasound gel was applied to ensure optimal acoustic contact. Under real-time ultrasound guidance, the shockwaves were focused on the gluteus medius tendon insertion at the greater trochanter. No local anesthetic was administered prior to treatment. All subjects underwent ESWT at an energy flux density of 0.20 mJ/mm^2^, 2400 pulses, and 6 Hz frequency once a week for three weeks. Patients were treated with 3 sessions of ESWT, once per week [21]. Clinical, ROM and US evaluations were performed at baseline (T0) when patients underwent the first ESWT treatment and at 6 months (T1) after the end of ESWT treatment.

### 2.3. Outcome Measures

**Figure 1:**
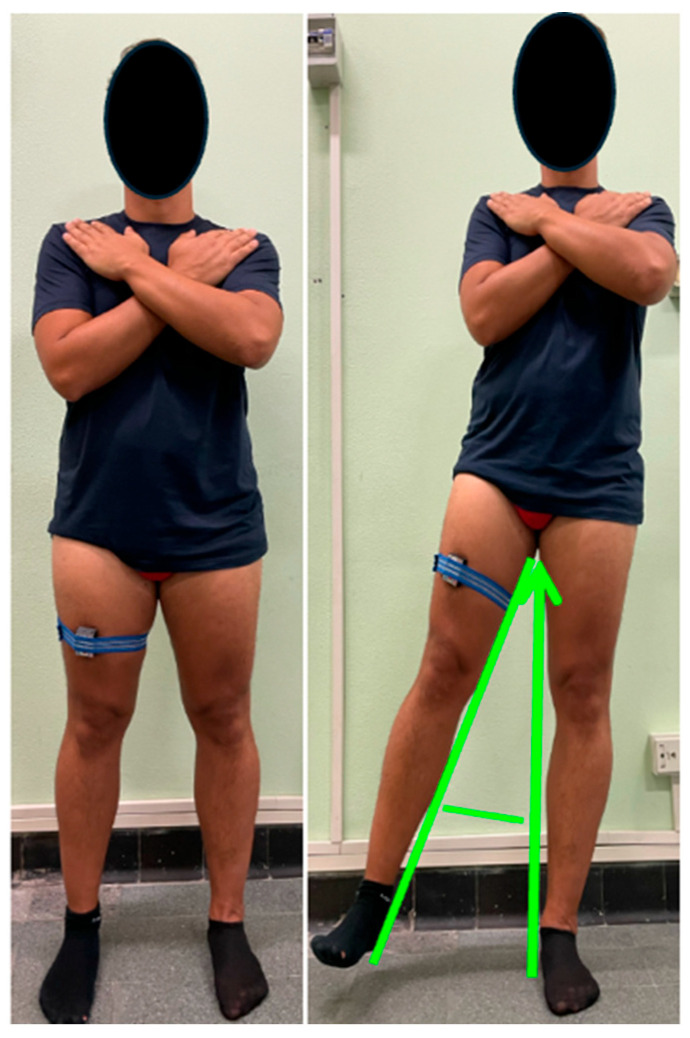
Standing hip abduction ROM assessment with IMU: neutral starting position (**left**) and peak abduction (**right**), with knee extended and no trunk or pelvic compensation.

Outcome measures were recorded by a physiatrist. The primary endpoint was the HHS, a standardized instrument assessing hip-related disability. It includes items addressing pain intensity, daily activity limitations during the preceding week, joint function, and range of motion. The total score ranges from 0 (severe disability) to 100 (normal hip function) [28]. Secondary outcome measures comprised the VAS for pain and the LEFS. The VAS consists of a 100 mm horizontal line anchored by “no pain” on the left (0 mm) and “worst imaginable pain” on the right (100 mm). Participants were asked to mark the point that best represented the intensity of pain experienced during their most painful movement. The score was quantified by measuring, in millimeters, the distance from the left anchor to the patient’s mark [29]. The LEFS is a self-administered questionnaire designed to evaluate functional status, monitor progress, and assess outcomes related to lower limb function. Scores range from 0 (severe functional limitation) to 80 (optimal function) [30,31,32]. The RM score was employed to assess pain and its impact on daily living activities. A score of 1 denoted an excellent quality of life, corresponding to the absence of symptoms, unrestricted walking ability without pain, and full satisfaction with the treatment results. A score of 2 indicated a good quality of life, characterized by the capacity to walk for more than one hour without pain, marked reduction of symptoms following therapy, and overall satisfaction with the outcome. A score of 3 reflected an acceptable quality of life, defined by limited walking endurance (less than one hour without pain), partial improvement of symptoms, more tolerable discomfort compared with baseline, and moderate satisfaction with the treatment. Finally, a score of 4 represented a poor quality of life, associated with severe pain during ambulation, persistence or worsening of symptoms after therapy, and dissatisfaction with the results [33]. Hip abduction ROM was assessed in a standing position using an IMU incorporating an accelerometer, gyroscope, and magnetometer (Fisiocomputer, Rome, Italy), exclusively on the ipsilateral (affected) side, following the approach used in previous studies on tendons pathology [34,35]. The sensor was secured with an elastic strap to the anterior aspect of the thigh, aligned with the midline of the femur, and maintained in a stable orientation throughout the measurement. Participants stood barefoot, with feet parallel and shoulder-width apart, and the trunk in a neutral upright position. To minimize compensatory movements, the upper limbs were crossed over the chest. The measurement was performed with the knee of the moving limb fully extended and the contralateral foot kept stationary and in full contact with the floor. From the neutral position, the participant lifted the limb laterally to the maximum tolerated ROM, avoiding lateral trunk lean or pelvic rotation. The peak abduction angle was defined as the maximal lateral deviation of the femoral axis relative to a vertical line drawn from the center of the pelvis (Figure 1). Each participant performed three repetitions of the movement, with a 10 s rest between trials, and the mean value of the three trials was used for analysis. The IMU was wirelessly connected to dedicated software that enabled real-time recording and processing of joint angles. An anatomical calibration in the neutral position was performed before data acquisition [36]. To minimize compensatory movements, all trials were continuously monitored by an experienced examiner through visual supervision, with particular attention to pelvic tilt and trunk inclination; trials showing evident compensations were repeated. Nevertheless, the absence of a dedicated pelvic sensor represents a methodological limitation.

**Figure 2:**
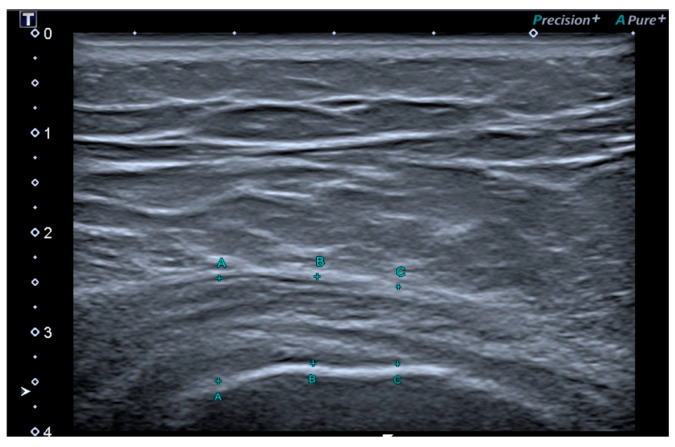
Measurement of Medius Gluteus tendon thickness in the longitudinal plane at three standardized points located 10, 15, and 20 mm above the reference point, defined as the lateral edge of the greater trochanter. The mean of the three measurements was used for analysis.

All ultrasonographic assessments were carried out by a musculoskeletal specialist with ten years of experience in diagnostic imaging and over two years of expertise in ultrasound elastography, who was blinded to treatment. Examinations were performed using a Toshiba Aplio 500 system (Canon Medical Systems Europe BV, Amstelveen, The Netherlands) equipped with a multi-frequency linear transducer operating between 5 and 18 MHz. Each participant was evaluated in the lateral decubitus position, with the affected side positioned upward and the hip maintained in slight flexion [37]. The GMT were examined with respect to morphology and echo texture in both longitudinal and transverse planes. Tendon thickness was assessed in the longitudinal plane at three distinct locations—10, 15, and 20 mm proximal to the reference point, defined as the lateral margin of the greater trochanter. The mean value of these three measurements (expressed in millimeters) was used for analysis (Figure 2). In every subject, the results were compared with the corresponding measurements obtained from the contralateral, asymptomatic side [7]. The contralateral side was clinically asymptomatic in all participants at the time of assessment, and no clinical evidence of subclinical alterations was observed.

**Figure 3:**
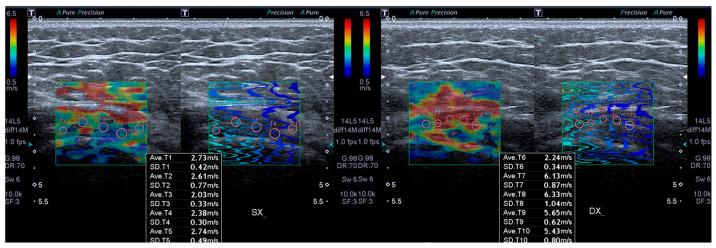
ROI measurements on shear-wave elastography velocity (SWEv expressed in m/s) images in a 46-year-old woman with GTPS. The left side of the image corresponds to the pathological (symptomatic) side, while the right side represents the healthy (asymptomatic) side.

SWE (m/s) was conducted immediately after the B-mode ultrasound assessment using an ultrasound unit equipped with elastography functionality. Shear wave velocity values were expressed in meters per second (m/s). The probe was positioned along the longitudinal axis of the gluteus medius tendon to obtain a clear view of the tendon fibers at their insertion. Within the insertional portion of the GMT, five circular regions of interest (ROI), each measuring 3 mm in diameter [38], were selected and evenly distributed to ensure representative sampling of the tendon tissue. Care was taken to exclude areas showing visible artifacts or irregularities on the propagation map. For each ROI, the system automatically generated a shear wave velocity measurement, and the mean of the five readings was used as the final elasticity value for the gluteus medius tendon (Figure 3).

### 2.4. Statistical Analysis

Sample size calculation was performed using G*Power (version 3.1.9.2, University of Kiel, Kiel, Germany). Based on published data on the HHS in gluteal tendinopathy [39], we assumed an expected pre–post improvement of 7 points, which meets the minimal clinically important difference (MCID) reported for this population (6.2 points). Using a paired *t*-test (one-tailed) with α = 0.05, power (1 − β) = 0.85, and an effect size dz = 0.46, the required minimum sample size was 35 patients. All statistical procedures were conducted using IBM SPSS Statistics, version 28 (IBM Corp., Armonk, NY, USA). Sample size calculation was based on the expected change in the Harris Hip Score; therefore, the study may not have been fully powered to detect changes in secondary outcomes such as SWE and IMU-based measurements, which should be interpreted accordingly. The Shapiro–Wilk test was employed to assess the normal distribution of continuous variables. Quantitative data are expressed as means ± standard deviations (SD), whereas qualitative data are presented as absolute frequencies and percentages. Descriptive statistics were calculated to summarize outcome measures both before and after treatment. Differences between pre- and post-intervention values were analyzed using paired-sample *t*-tests. To explore the relationships between range of motion (ROM), ultrasound parameters, and clinical scores, correlation analyses were performed. Pearson’s correlation coefficient was applied to evaluate linear associations among continuous variables. Correlation strength was interpreted as follows: coefficients between 0.3 and 0.7 indicated a moderate positive relationship, while values between −0.3 and −0.7 reflected a moderate negative relationship [40].

The primary outcome was expressed as Delta Harris Hip Score (ΔHHS), calculated as the difference between post-treatment (T1) and baseline (T0) Harris Hip Score values. A univariate General Linear Model (GLM) was performed with ΔHHS as the dependent variable, and age, BMI, tendon thickness, baseline SWEv, baseline VAS, and gender as predictors. Statistical significance was set at *p* < 0.05.

## 3. Results

### 3.1. Patient Demographic and Clinical Characteristics

**Figure 4:**
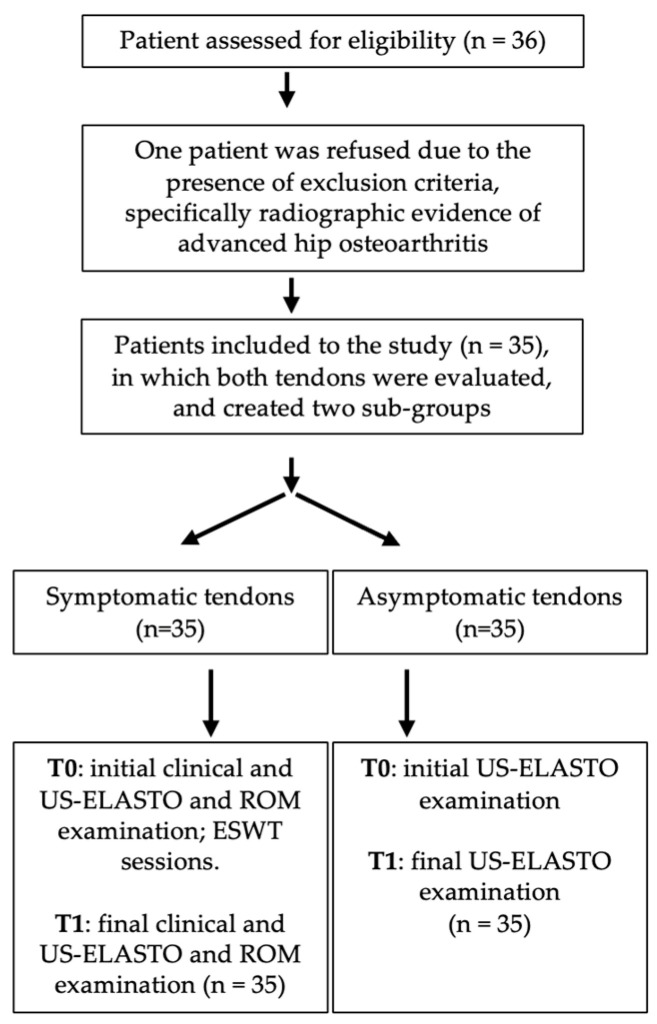
Flowchart illustrating the study course.

Patient recruitment and data collection were performed between March 2024 and May 2025 in Italy. The course of the study is illustrated in Figure 4. A total of 36 patients were assessed for eligibility, and 35 (97.2%) were included in the study based on inclusion and exclusion criteria. From the study population, 5 were males and 30 were females (mean age 67.3 ÷ 9.9), so in total 70 tendon samples (35 symptomatic, 35 asymptomatic) were evaluated. One patient was not included for following reason—reported previous tendon injury and two reported other interventions targeting GMT less than 3 months prior to the baseline examination. During the study, no patient withdrew. The baseline and follow-up demographic and clinical characteristics of patients are reported in Table 1.

### 3.2. Outcome Measures Results

At baseline, the affected side showed a thickened GMT comparing with healthy side (9.6 ± 1.5 vs. 8.1 ± 1 mm) (*p* < 0.05). Table 2 shows the B-mode and elastographic results at baseline and after ESWT treatment at T1. Six months after ESWT, the thickness of symptomatic side decreased from the baseline (9.6 ± 1.5 vs. 8.6 ± 1.1 mm) (*p* < 0.05). Six months after ESWT, the affected side showed a thickened GMT comparing with healthy side (8.6 ± 1.1 vs. 8.1 ± 1 mm) (*p* < 0.05).

At baseline, the affected side showed lower GMT SWEv expressed in m/s than the healthy side (1.8 ± 0.3 vs. 4.2 ± 0.7 m/s) (*p* < 0.05). Six months after ESWT, the SWEv of affected side increased from the baseline (3.2 ± 0.8 vs. 1.8 ± 0.3 m/s) (*p* < 0.05). Six months after ESWT, the affected side showed lower GMT tendon SWEv than the healthy side (3.2 ± 0.8 vs. 4.2 ± 0.7 m/s) (*p* < 0.05).

The baseline average for Hip abduction, HHS, VAS, LEFS and RM were, respectively, 33.6° with ±1.8° SD, 61.6 points with ± 10.1 points SD, 6.4 points with ±1.4 SD, 46.5 points with ±10.9 points SD and 3.2 points with ±0.7 SD (Table 3). At T1, the Hip abduction, HHS, VAS LEFS and RM had an average, respectively, of 35.2° with ±2° SD, 78.7 points with ±14.1 points SD, 3.8 points with ±2.1 SD, 55.3 points with ±9.4 points SD and 2.1 points with ±0.8 SD.

When considering hip abduction ROM, a significant negative correlation was observed between abduction at T1 and VAS at T1 (r = −0.424; *p* = 0.011). At baseline, abduction ROM was negatively correlated with VAS (r = −0.428; *p* = 0.010) and RM score (r = −0.346; *p* = 0.042), and positively correlated with LEFS score (r = 0.366; *p* = 0.031), suggesting that greater initial ROM was associated with lower pain, better function, and a more favorable clinical status.

For elastographic measures, a significant negative correlation was found between SWEv and VAS at T1 (r = −0.600; *p* < 0.05), and a significant positive correlation was found between SWEv and HHS at T1 (r = 0.400; *p* < 0.05), suggesting that higher tendon stiffness values at follow-up were associated with lower pain and better hip function.

In the univariate GLM including age, BMI, tendon thickness, baseline SWEv, baseline VAS, and gender as predictors of ΔHHS, the overall model was significant (F = 2.754, *p* = 0.031, adj. R^2^ = 0.236). Lower baseline SWEv (B = −13.8, *p* = 0.047, η^2^ = 0.134) and higher baseline VAS score (B = +3.77, *p* = 0.014, η^2^ = 0.199) were associated with greater improvements in HHS.

Throughout the study period, no significant adverse effects related to the therapies were observed or reported by the participants.

## 4. Discussion

This study provides the first in vivo demonstration of tendon remodeling after ESWT using an integrated biosensing approach that combines SWE expressed in m/s and IMU. However, in the absence of a control, sham, or comparative treatment group, causal inference remains limited, and the observed improvements cannot be unequivocally attributed to ESWT alone, particularly given the known placebo-responsive components associated with this intervention. A further limitation is the absence of intra- and inter-observer reliability assessment for SWE and IMU measurements, which are partly operator- and device-dependent. This multimodal framework enables simultaneous quantification of tendon mechanical properties and functional mobility, overcoming the limitations of traditional, subjective clinical scales. By merging imaging and motion sensing technologies, the present study introduces an objective, quantitative model for monitoring musculoskeletal recovery after ESWT.

At baseline, symptomatic GMT exhibited markedly reduced SWE velocities expressed in m/s compared with the contralateral healthy side, reflecting decreased stiffness and elasticity associated with degenerative tendinopathy. Following ESWT, SWE velocity significantly increased, while tendon thickness decreased, indicating a partial restoration of structural integrity reflecting reduced intratendinous edema. Given the limited availability of longitudinal shear-wave elastography studies in greater trochanteric pain syndrome, comparisons with elastographic data from other tendons are necessary. In this context, the present findings are consistent with previous elastographic studies of the Achilles and supraspinatus tendons [41,42], supporting the role of SWE as a reliable imaging biomarker of intratendinous tissue mechanical properties. Tendon stiffness measured by SWE reflects a complex biomechanical phenotype influenced by both local tissue structure and systemic biological factors. A previous study on the patellar tendon has shown that metabolic variables, such as low-density lipoprotein levels, may modulate tendon mechanical properties independently of body mass index, supporting the biological sensitivity of elastographic measurements [43,44]. The persistence of slightly greater thickness on the treated side may indicate that complete structural normalization requires a longer follow-up period or additional interventions. From a tissue biology perspective, it can be hypothesized that the SWE-derived changes observed in this study might reflect processes such as collagen turnover, extracellular matrix remodeling, or vascular adaptations, which have been previously described in association with ESWT, although no causal inferences can be drawn from the present observational design [45,46,47].

In addition to imaging findings, the IMU-based assessment revealed significant improvement in hip abduction ROM after therapy; the negative correlations between hip abduction and pain intensity, and the positive association with LEFS, indicate that wearable sensor data reliably captured functional recovery. Unlike conventional goniometric measurements, IMU-based sensing provides high-resolution, reproducible data independent of operator bias, allowing quantitative evaluation of dynamic performance [48]. As previously observed, these results support the concept of motion sensing as a functional biosensor capable of continuously monitoring motor outcomes during recovery [49,50].

Similarly, improvement in tendinopathy has been associated with both pain reduction and recovery of mobility in the affected muscle–tendon unit [51,52,53]. In the present study, the significant, albeit modest, increase in hip abduction ROM from baseline to follow-up suggests a partial restoration of functional capacity of the gluteal muscles and tendons following ESWT. This finding aligns with previous reports showing that targeted interventions for GTPS, such as exercise therapy or shockwave application, can yield measurable gains in mobility alongside improvements in patient-reported outcomes [4,17,54]. The recovery of hip abduction ROM may reflect decreased pain inhibition, improved neuromuscular activation, and a gradual reversal of movement adaptations adopted to avoid loading the affected tendon. This interpretation is supported by the observed associations between greater ROM and lower pain intensity, better functional scores, and more favorable clinical ratings both at baseline and at the end of follow-up, indicating a consistent link between hip mobility and overall clinical status.

As observed in past studies, the integration of multidomain data allows for a comprehensive characterization of tendinopathies [55,56,57,58], and the integration of SWE and IMU data offers a multidimensional biosensing framework to characterize tendon healing from both structural and functional perspectives. While SWE quantifies mechanical properties and tissue remodeling [59,60,61], IMU-derived motion metrics reflect neuromuscular performance and pain inhibition patterns [62]. Their combined use generates complementary biosignals that can serve as objective endpoints for clinical trials and precision rehabilitation monitoring. This dual-sensor approach aligns with emerging trends in personalized medicine, where multimodal biosensing is used to guide and optimize therapeutic strategies [63].

In another study evaluating pain reduction using the VAS in patients with GTPS treated with focused ESWT, a magnitude of VAS improvement similar to that observed in our cohort was reported, supporting the robustness and clinical consistency of the pain-related outcomes in the present study. By contrast, other objective outcomes assessed in our investigation—such as tendon mechanical properties and quantitative motion parameters—were not evaluated in previous studies on this topic [21].

Univariate GLM analysis identified lower baseline SWEv and higher baseline VAS scores as predictors of greater functional improvement after ESWT, suggesting that baseline mechanical and symptomatic profiles can influence treatment response [64]. These findings are consistent with previous elastography studies showing that tendons with lower stiffness values have greater remodeling potential under mechanical stimulation [65,66].

## 5. Limitations and Future Perspectives

This investigation has some limitations. The study population consisted predominantly of older adults; therefore, the findings may not be directly generalizable to younger individuals or athletic populations with GTPS. Intra-observer reliability for shear-wave elastography and tendon thickness measurements was not formally assessed, which represents a limitation of the study. Additionally, the absence of a control group and randomization limits the ability to draw causal inferences regarding the observed effects. The relatively small sample size and short-term follow-up prevent conclusions on long-term tendon adaptation. Future research should validate these results in larger, randomized cohorts and explore advanced signal integration approaches using artificial intelligence or machine learning for predictive modeling [67,68].

## 6. Conclusions

In this study, wearable IMU provided quantitative evidence of improved hip abduction ROM following ESWT in patients with GTPS. The observed correlations between ROM, pain reduction, and functional recovery highlight the potential of motion sensing as a functional biosensor capable of capturing dynamic improvements during rehabilitation. This wearable, operator-independent approach allows objective tracking of motor performance, complementing traditional clinical assessments. In parallel, SWE expressed in m/s is a significant indicator of tissue mechanical properties of the gluteus medius tendon, with increased elasticity and reduced thickness after treatment. The association between SWE values and clinical outcomes supports its role as an imaging biomarker of tendon recovery. Together, IMU- and SWE-derived metrics provide complementary insights into the functional and structural dimensions of tendon healing. This dual-sensor framework represents a step toward integrating quantitative motion analysis and imaging assessment for more precise and individualized therapies. However, further prospective evaluations on a larger population and randomized controlled trials are warranted to confirm these promising results.

## Figures and Tables

**Table 1:** Comparison of variables and outcomes measures before and after treatment. Values are reported as mean ± standard deviation for continuous variable and as distribution for categoric variable.

Variable	Value
Age (years)	67.3 ± 9.9
Gender (Male/Female)	5/30
BMI	26.4 ± 4.9
HHS T0	61.6 ± 10.1
HHS T1	78.7 ± 14.1
VAS T0	6.4 ± 1
VAS T1	3.8 ± 2.1
LEFS T0	46.5 ± 10.9
LEFS T1	55.3 ± 9.4
RM T0	3.2 ± 0.7
RM T1	2.1 ± 0.8
Hip Abduction T0	33.6 ± 1.8
Hip Abduction T1	35.2 ± 2
Thickness affected side T0	9.6 ± 1.5
Thickness affected side T1	8.6 ± 1.1
Thickness healthy side T0	8.1 ± 1
SWEv affected side T0	1.8 ± 0.3
SWEv affected side T1	3.2 ± 0.8
SWEv healthy side T0	4.2 ± 0.7

BMI: Body Mass Index; VAS: visual analogue scale; HHS: Harris Hip Score; LEFS: Lower Extremity Functional Scale; RM: Roles and Maudsley score; SWEv: Shear wave elastography velocity (m/s).

**Table 2:** B-mode and elastographic evaluation at baseline and after ESWT treatment.

	Symptomatic GMT T0	Symptomatic GMT T1	Healthy GMT T0	*p*-Value
Gluteus Medius Tendon Thickness	9.6 ± 1.4	8.6 ± 1.1	8.1 ± 1	<0.05
SWEv (m/s)	1.8 ± 0.3	3.2 ± 0.8	4.2 ± 0.7	<0.05

Data are presented as mean ± standard deviation (SD). T0: baseline, T1: 6 months after treatment. GMT: Gluteus Medius Tendon; SWEv: Shear Wave Elastography velocity.

**Table 3:** Mean and standard deviation of Visual Analog Scale (VAS) Scores, Roles & Maudsley (RM), Harris Hip Score (HHS) and Lower Extremity Functional Scale (LEFS) of patients with greater trochanteric pain syndrome after treatment with extracorporeal shockwave therapy (ESWT).

	Hip Abduction	VAS Score	Harris Hip Score	Lower Extremity Functional Scale	Roles and Maudsley
Baseline T0	33.6 ± 1.8	6.4 ± 1.4	61.6 ± 10.1	46.5 ± 10.9	2.3 ± 0.6
Follow-up T1	35.1 ± 2	3.9 ± 2.1	78.7 ± 14	55.2 ± 9.4	1.5 ± 0.8

## Data Availability

The datasets used, and data analyzed during the current study will be made available upon reasonable request to the corresponding author (G.S.).
